# Mosaic Turner Syndrome With 45,X/46,XY Mosaicism and Apparent Absent Uterus

**DOI:** 10.7759/cureus.14816

**Published:** 2021-05-03

**Authors:** Alya Alhajjaj, Sarraa A Altarouti, Fatimah Alkhabbaz

**Affiliations:** 1 Internal Medicine and Endocrinology, Qatif Central Hospital, Qatif, SAU; 2 Internal Medicine, Qatif Central Hospital, Qatif, SAU

**Keywords:** turner, 45x/46xy, absent uterus, mgd

## Abstract

Turner syndrome (TS) is a relatively common chromosomal abnormality in females. Short stature, gonadal dysgenesis, and somatic dysmorphic features are the characteristic features of the syndrome. The chromosomal abnormalities of TS are highly variable; 45,X/46,XY mosaicism accounts for 10-12% of cases of Turner syndrome. Despite the presence of hypogonadism, affected females typically have a uterus. Here, we report the case of a 22-year-old female who presented at 15 years of age with primary amenorrhea. She was diagnosed with Turner syndrome mosaicism with a karyotype of 45,X/46,XY. Her pelvic imaging showed an absent uterus and ovaries. Due to the presence of a Y chromosome, she underwent prophylactic gonadectomy. Histopathology of her removed gonads confirmed the diagnosis of mixed gonadal disorder. She was started on estrogen replacement. Four years after treatment, she developed her menses. Her repeated pelvic magnetic resonance imaging showed the presence of a small uterus.

## Introduction

Turner syndrome (TS) is characterized by a complete or partial loss of the second X chromosome in female patients. Its estimated prevalence is 1:2500-3000 live-born females [1]. Short stature, gonadal dysgenesis, and specific somatic dysmorphic features are the characteristic features of the syndrome [2].

Turner syndrome is diagnosed in females based on their clinical presentations and their chromosomal abnormalities. It has highly variable chromosomal abnormalities with a different reported prevalence between studies. Baena et al. reported that the typical karyotype for TS (45,X) found in 81.6% of patients followed by variable types of mosaicism in 16.8% of the patients [3]. In cases with mosaicism, 45,X cell line has one or more other cell lines with structurally abnormal or a complete sex chromosome (Y or X). Structural abnormalities of the sex chromosome (X) can be due to duplication of the long arm to form isochromosome, deletions of the long arm p or short arm q, or formation of a ring. Some TS females may carry extra cell lines, with the Y chromosome (45,X/46,XX; 45,X/46,XY) [4].

The main presentation of patients with 45,X/46,XY mosaicism is mixed gonadal dysgenesis (MGD), but they may have different phenotypes depending on the percentage distribution of mosaicism in gonadal tissues and blood. These phenotypes include normal female, TS, genital ambiguity, and male phenotype [5].

We present a case of TS with an MGD genotype and a female phenotype. Her uterus was not seen by her initial imaging studies. After being on estrogen replacement for four years, she developed a small uterus with regular menses.

## Case presentation

A 22-year-old Saudi female presented at 15 years of age (March 2013) with primary amenorrhea. She was born through normal spontaneous vaginal delivery with normal developmental and mental milestones. She stated that she was shorter than her siblings. She denied any medical illness. Her school performance was excellent.

Her physical examination revealed a height of 134 cm, which was below the third percentile, and a body mass index of 36 kg/m2. There were no clear dysmorphic features suggestive of TS. Breasts were Tanner stage V. Her pelvic examination showed pubic hair Tanner stage III with normal external female genitalia. The systemic examination was unremarkable except for acanthosis nigricans on the nape of the neck.

Her investigations showed normal complete blood count, basic metabolic panel, and echocardiogram. Her bone age was delayed by two years from her chronological age. Her hormonal profile showed hypergonadotropic hypogonadism with Luteinizing hormone (LH) of 33.57 mIU/L (normal, 0.11-29.4), follicle-stimulating hormone (FSH) of 65.12 mIU/L (normal, 3-12.5), and estradiol of 14.7 pg/ml (normal, 40-110). Her thyroid function test was normal. Her pelvic ultrasound (U/S) showed a vaginal pouch of 2.3 cm, uterus and ovaries were not visualized, and right ectopic kidney. Her pelvic magnetic resonance imaging (MRI) showed similar findings to her pelvic U/S. There was no evidence of osteoporosis on her bone mineral density (DEXA, dual-energy X-ray absorptiometry) scan. The karyotype analysis was sent for the patient and repeated three times (standard karyotype and fluorescence in situ hybridization [FISH] method) and showed 45X (20), 46XY (5) mosaic TS. 

**Figure 1:**
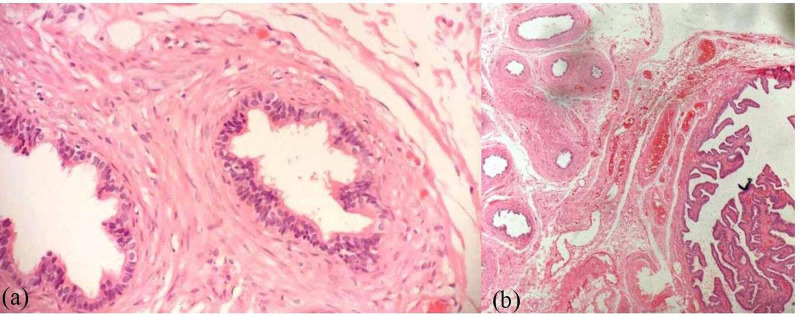
Histopathology evaluation (a) right gonad showed rete testis tissue and ductuli efferentes, (H&E, x20); (b) left gonad showed fallopian tube, (H&E, x20) H&E: hematoxylin-eosin stain

She underwent bilateral laparoscopic gonadectomy in December 2013. Histopathological evaluation showed rete testis tissue and ductuli efferentes on the right gonad (Figure 1a), while the left gonad showed fallopian tube (Figure 1b).

**Figure 2:**
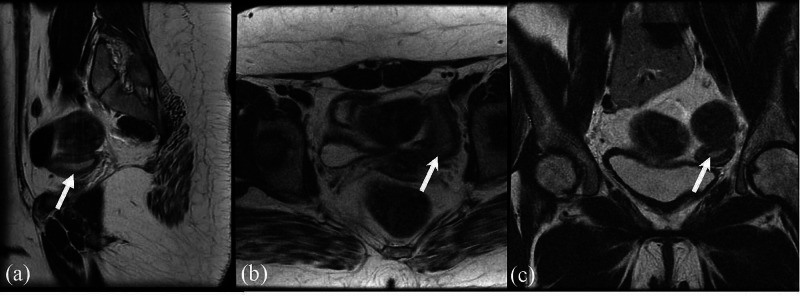
Repeated MRI of the uterus, after four years of management with OCP (a) sagittal T2 shows the uterus (arrow); (b) axial T2 shows the uterus (arrow); (c) coronal T2 shows the uterus (arrow) OCP: oral contraceptive pill

The patient was started on estrogen replacement in the form of oral contraceptive pills (OCP). Four years after treatment with OCP, the patient started to have withdrawal bleeding. Pelvic MRI (Figure 2) was repeated and it showed a small uterus measuring 4.9 x 1.7 x 3.1 cm. Currently, she still has regular menses and her final height is 152 cm.

## Discussion

Our patient is a case of TS with 45,X/46,XY mosaicism with MGD. Mixed gonadal dysgenesis is defined as having a differentiated testis on one side with a contralateral streak testis or streak gonad. Although TS is associated with a variable sex chromosome mosaicism, 45,X/46,XY is the most common mosaicism [6]. The 45,X/46,XY genotype affects nearly 10-12% of TS cases [7]. It may present with variable phenotypes [5]. Our patient was phenotypically female.

The presence of different histological abnormalities including ovotestes, streak gonads, and other abnormalities has been investigated in several studies including a multicenter study involving 48 patients with 45,X/46,XY mosaicism [8]. The histopathology of our patient’s removed gonads showed mixed gonadal tissues. 

The presence of Y chromosome in TS increases the risk of malignancy particularly gonadoblastoma by 7-10%. Consequently, prophylactic gonadectomy at the time of diagnosis is highly recommended in female patients who got Y chromosome material in their karyotype [1]. In a study of 107 TS patients, six patients with an identified Y chromosome on PCR (polymerase chain reaction) study underwent gonadectomy. Two cases were found to have gonadoblastoma, accordingly, the incidence of gonadoblastoma was 33% [9]. Our patient underwent bilateral laparoscopic gonadectomy in the same year of the diagnosis.

Our patient had an apparent absent uterus on her initial pelvic imaging. Muntaj et al., in their case series, reported a 15 years old girl with MGD and an absent uterus as our patient [4]. Despite the typical presentation of hypergonadotropic hypogonadism in TS, the uterus is usually present, but it may be small [10]. Our patient developed an apparent absent uterus after four years of estrogen replacement which was clear on her repeated MRI.

After an extensive literature review, we came across two reported cases with similar apparent absent uterus with uterine growth after estrogen therapy. The first case was a 17-years-old girl with TS of karyotype 45,X. The patient had an absent uterus and ovaries which was confirmed by MRI. After six months of estrogen replacement, the patient developed a small uterus measuring 4.5 cm in length [11]. The second case was a 14-years-old female with TS (karyotype 45,X) who had a complete absence of uterus and ovaries. After eight months of estrogen therapy, the patient developed a uterus measuring 5.7 x 1 x 1.9 cm on her pelvic U/S [12]. Both of these patients had 45,X genotype, in contrast to our patient who had 45,X/46,XY mosaicism.

## Conclusions

A combination of absent uterus and Turner syndrome has never been reported before. A finding of absent uterus on imaging in these hypoestrogenic patients should be interpreted carefully. Follow-up imaging in these patients is recommended after estrogen therapy.
